# *TTN* Variants Are Associated with Physical Performance and Provide Potential Markers for Sport-Related Phenotypes

**DOI:** 10.3390/ijerph191610173

**Published:** 2022-08-17

**Authors:** Agata Leońska-Duniec, Małgorzata Borczyk, Marcin Piechota, Michał Korostyński, Andrzej Brodkiewicz, Paweł Cięszczyk

**Affiliations:** 1 Faculty of Physical Education, Gdansk University of Physical Education and Sport, 80-336 Gdansk, Poland; 2 Laboratory of Pharmacogenomics, Department of Molecular Neuropharmacology, Maj Institute of Pharmacology, Polish Academy of Sciences, 31-343 Kraków, Poland; 3 Department of Pediatrics, Child Nephrology, Dialisotherapy and Management of Acute Poisoning, Pomeranian Medical University, 70-204 Szczecin, Poland

**Keywords:** titin, muscle fibers, elite athletes, sequencing, candidate gene, sport genetics

## Abstract

*TTN* encodes the third myofilament, titin, which plays structural, mechanical, regulatory, and developmental roles in sarcomeres. The aim of this research was to determine the interaction between novel and previously described *TTN* variants and athletic performance, as well as competition level, in Caucasians. Firstly, 100 athletes and 47 controls were recruited, and whole-genome sequencing was performed. Secondly, 348 athletes (108 endurance, 100 sprint/power, 140 mixed-sport athletes) and 403 volunteers were included, and real-time PCR was performed. We found a significant overrepresentation of the rs10497520 CT and TT genotypes in the sprint/power athlete group (95% CI, 1.41–3.66, *p* = 0.0013). The rs10497520 T carriers were 2.17 times more likely to become sprint/power athletes (95% CI 1.35–3.49, *p* = 0.0021). We also found that the likelihood of having the TT genotype was higher for the highly elite and sub-elite sprint/power athletes. Possessing at least one TAA (rs10497520, rs55837610, rs72648256) haplotype resulted in an increase in the log-odds ratio by 0.80 (*p* = 0.0015), 1.42 (*p* = 0.003), and 0.77 (*p* = 0.044) for all, highly elite, and sub-elite sprint/power athletes, respectively. We demonstrated that harbouring the rs10497520 T allele, individually and in a haplotype combination, increased the chance of being an elite sprint/power athlete, indicating that this allele may be favourable for sprint/power performance.

## 1. Introduction

In the 1950s, the filamentous nature of the sarcomere was established, and muscle contraction was considered in terms of the interaction between two contractile filaments, actin and myosin (referred to as the sliding filament theory) [1,2]. The discovery of a third filament, titin, also known as a connectin, challenged the previous concepts and aroused the interest of scientists in explaining its role in the development of the structural and functional properties of sarcomeres and muscle as a whole [3,4,5].

Titin is the largest known protein and has a very complex structure, which extends from the Z-disc to the M-line. Four structurally and functionally distinct regions with numerous sequence domain repeats can be distinguished: (i) an amino-terminal Z-disc region, which acts as an anchor, embedding titin to the Z-disc through binding to proteins such as α-actinin and functioning as a part of the mechanical stretch sensor machinery; (ii) a middle I-band region, which acts as a molecular spring, providing myocyte elasticity and maintaining the Z- and M-line connections during muscle elongation and contraction; (iii) an A-band region, which is important for thick filament termination, interaction with myosin-binding protein C, and modification of the thick filament length; and (iv) a carboxy-terminal part spanning the M-line, which is involved in various signalling pathways [6,7,8]. Detailed visualisations of the domain layout of the protein and its position in the sarcomere are available in multiple excellent reviews about the protein [6,7,9]. Thus, titin performs a variety of functions in muscle contraction and force production, including structural and developmental roles in the organisation of sarcomeres, mechanical roles in passive and active force regulation in cardiac and skeletal muscles, and serving as a sensory and signalling mediator [9,10,11,12,13,14]. Undoubtedly, we are only in the early stages of understanding the role of titin in the formation of muscle and its properties [15].

The giant *TTN* gene (2q31.2) encoding titin consists of 365 exons and transcribes an mRNA over 100 kb long. Due to extensive mRNA splicing, human *TTN* generates seven isoforms with different physiological properties: the longer isoforms are more elastic, while the shorter isoforms are stiffer [6,16,17,18]. The main skeletal muscle isoform is N2A, which is the longest known isoform, expressing 312 exons but lacking exon 49, which encodes the N2B domain in the I-band region. In contrast, the cardiac-specific isoforms are shorter, and two major isoforms, N2B (191 exons) and N2BA (313 exons), have been identified. Depending on muscle type and developmental stage, different lengths of these titin isoforms are produced [6,8,19]. *TTN* polymorphisms may affect the contractile characteristics of titin filaments, which may be a favourable or unfavourable factor for muscle performance [8,20]. The *TTN* gene has been described as a major human disease gene [21]. Recently, *TTN* gene variants have been linked to numerous inherited diseases of skeletal and cardiac muscle [21,22,23,24]; however, their relationship with physical performance, athletic status, and injury risk is almost unknown.

Subsequent research has confirmed the key role of titin in the development of crucial skeletal and cardiac muscle functions, such as strength and stiffness, and highlighted its role in sports performance [6,20,25,26], and in all probability as argued by Perrin et al. (2017) in the risk of injury [27]. The best-known variation within the *TTN* gene is the C>T polymorphism (rs10497520), which results in a lysine (Lys) transforming into a glutamic acid (Glu), which may affect the variability within the isoforms’ expression in muscle tissue [20]. To date, this polymorphism has been associated with differences in the maximal oxygen consumption (VO_2_max) training response [25] and muscle fascicle length [20].

These studies have suggested that the *TTN* gene may be a very promising candidate gene for physical performance and injury risk. However, whole *TTN* gene analysis is very complicated due to its large size and complex structure. As next-generation sequencing (NGS) has become widely available, it has recently become possible to examine the *TTN* gene structure and identify specific mutations that influence physical performance and human health. To date, more than 120 *TTN* polymorphisms related to the human phenotype have been identified [6]. However, *TTN* gene sequencing in varied populations, as well as the isoform diversity and reference sequences used, underline the problems in assessing the significance and relationship between the sequence changes and the observed phenotype. Thus, the role of *TTN* mutations in modifying parameters associated with sports skills is still unexplained. Therefore, the aim of this study was to determine the interaction between novel (rs72648256 and rs55837610) and previously described (rs10497520) allelic variants, genotypes, and haplotypes located in the *TTN* gene and athletic performance and competition level in the Caucasian population. Taken together, we can verify the usefulness of the *TTN* gene as a genetic marker for sports skills, which may underpin differences in the potential to be an elite athlete.

## 2. Materials and Methods

### 2.1. Ethics Statement

The experimental protocols were conducted according to the World Medical Association Declaration of Helsinki, the Strengthening the Reporting of Genetic Association studies (STREGA) statement, and ethical standards in sports science research. All procedures were approved by the Ethics Committee at The District Medical Chamber in Gdansk (KB-8/19). Written informed consent was obtained from all individual participants.

### 2.2. Participants

In the first part of the experiment involving whole-genome sequencing (WGS), the study group consisted of 100 male elite sprint/power (n = 52) and endurance (n = 48) athletes (age: 23.5 ± 5.9 years) of the highest nationally-competitive standards (classification was based on scoring tables, e.g., IAAF, FINA, or receiving a medal in the national championships, or participation in international competition at the European or World Championships). Athletes with personal best results ranking them in the top 100 in a particular sports discipline in the world or in Europe were included in the study group. As the aim of this part of the study was to determine genetic variants associated with overall physical performance, all athletes were considered as a single group.

The control group consisted of 47 healthy individuals with no pairs with kinship above 0.125 (kinship was assessed with hl.pl_relate method, kinship metric scale 0–5) (age: 22.4 ± 6.3 years). The inclusion criteria for volunteers were no medical history of any cardiorespiratory diseases, and not participating in professional sport training.

The second part of our study aimed to assess the association of selected *TTN* variants with sport-related phenotypes, and 348 Polish athletes (age: 27.8 ± 7.1 years) who competed in national and international events were involved. The athletes were stratified into three groups according to values of relative anaerobic/aerobic energy system contribution, time of competitive exercise performance, and intensity of exertion in each sport: endurance (n = 108), sprint/power (n = 100), and mixed-sport (n = 140) athletes. The athletes in these groups were divided into subgroups according to their competition level: highly elite (gold medalists in the World and European Championships, World Cups, or Olympic Games), elite (silver or bronze medalists in the World and European Championships, World Cups, or Olympic Games), and sub-elite (participants in international competitions). The breakdown among groups was as follows: high elite (n = 19), elite (n = 45), sub-elite (n = 36) sprint/power athletes; high elite (n = 19), elite (n = 47), sub-elite (n = 42) endurance athletes; and high elite (n = 17), elite (n = 63), sub-elite (n = 61) mixed-sport athletes.

Non-training unrelated students (n = 403) from the Gdansk University of Physical Education and Sport (age 22 ± 3.4 years) were included in the control group.

Athletes in the first and second part of the study, as well as controls, were of Caucasian origin.

We performed sample size calculations to assess the power of the genotyping study, assuming the following parameters: power 90%, effect size 25%, and alpha 0.05. The results indicated a minimum group size of 329 participants per group.

### 2.3. WGS and Data Processing

WGS was performed externally by the BGI company using the following parameters: paired reads of 150 bps, at least 90 GB of data per sample with 300 M reads and 30× coverage. Fastq files were processed with Intelliseq Germline Pipeline 1.8.3 (https://gitlab.com/intelliseq/workflows, accessed on 9 December 2021) built with Cromwell (https://cromwell.readthedocs.io/en/stable/, accessed on 9 December 2021). Within the pipeline, Fastq file quality was assessed with fastQC. Fastq files were then aligned to the Broad Institute Hg38 Human Reference Genome with GATK. Duplicate reads were removed with Picard, and base quality Phred scores were recalibrated using GATK’s covariance recalibration. Variants were called with GATK HaplotypeCaller, and genomic variant calling files (gvcf) were obtained. Files were then imported to Hail (0.2.62, https://hail.is/ (accessed on 9 December 2021)).

### 2.4. WGS Data Filtering and Annotation

All analyses described below were performed with Hail (0.2.62, https://hail.is/ (accessed on 14 December 2021)). Detailed information about file preparation, annotation and filtering has been provided to the project Github repository (https://github.com/ippas/imdik-zekanowski-sportwgs, accessed on 9 December 2021). Briefly, all gvcf were combined into a sparse matrix table and genotyped. Multiallelic variants were split. The Vcf file with all alternate allele calls in the analysed cohort was filtered to exclude repeated and low-complexity sequences (UCSCRepeatMasker track). Only loci with more than 90% gnomAD v3 samples with a DP of >1 were kept for further analysis. Variants were annotated with gnomAD (v3; https://gnomad.broadinstitute.org/ (accessed on 14 December 2021)). One sample was excluded from the analysis based on PCA of 0.1% of genotypes.

### 2.5. Variant Filtering

To narrow the list of analysed genotypes and to exclude common mutations, a minor allele frequency (MAF, based on gnomAD v3 non-Finnish Europeans) filter of 0.5% was used. Each of the genotypes was tested with Fisher’s exact test to find over- or under-represented variants in elite sportsmen.

### 2.6. SNPs Genotyping

Genomic DNA was extracted from buccal cells with a GenElute Mammalian Genomic DNA Miniprep Kit (Sigma, Steinheim, Germany) according to the manufacturer’s protocol. All samples were genotyped in duplicate using an allelic discrimination assay on a C1000 Touch Thermal Cycler (Bio–Rad, Feldkirchen, Germany) instrument with TaqMan^®^ probes. To differentiate the *TTN* rs10497520, rs72648256, and rs55837610 alleles, TaqMan^®^ Pre-Designed SNP Genotyping Assays (Applied Biosystems, Waltham, MA, U.S.A.) (assay ID: C___1958912_10, C__96796333_10, C__88429447_10, respectively) including fluorescently labelled (FAM and VIC) minor groove binder (MGB) probes and primers were used.

### 2.7. Statistical Analyses

Hardy–Weinberg equilibrium was evaluated using the exact test. An association analysis between a single SNP and dependent categorical variables was conducted under four different genetic models (inheritance patterns): co-dominant, dominant, recessive and over-dominant, whenever possible. The odds ratio and 95% confidence interval (taking the most frequent homozygous genotype as the reference) were calculated. Haplotypes were inferred using an expectation–maximisation algorithm. In only one case was the haplotype ambiguous. Unambiguous haplotypes were used in further analysis, and a pair of haplotypes were assigned to each individual. Only haplotypes with frequencies >1% were considered. Haplotypes were tested for association under the assumption of a dominant model using a general linear model. SNPassoc and haplo.stats package for R (R Core Team (2020), R: A language and environment for statistical computing, R Foundation for Statistical Computing, Vienna, Austria, URL https://www.R-project.org/ (accessed on 27 January 2022)) were used for single SNP and haplotype analysis, respectively. *p* values < 0.05 were considered significant.

## 3. Results

Three polymorphisms were selected for the study: rs10497520 (Lys1201Glu) based on a literature review, and two rs55837610 (Ile23902Thr) and rs72648256 (Asp31617Glu) based on the WGS results. All polymorphisms were in Hardy–Weinberg equilibrium (*p* = 0.600, *p* = 1.0, *p* = 1.0, for *TTN* rs10497520, rs55837610, and rs72648256, respectively) in control individuals. rs10497520 deviated from expectations in the sprint/power group (*p* = 0.037).

Comparisons of all athletes (sprint/power, endurance, and mixed-sports athletes) with controls revealed no significant associations (Table 1), regardless of assumptions about the genetic model of a trait. When athletes were stratified by sport discipline into sprint/power, endurance, and mixed-sports groups (Table 2, Table 3 and Table 4), we found a significant over-representation of the rs10497520 CT and CT-TT genotypes in the sprint/power athlete group (Table 2). The genotypic odds ratio (OR) for genotype CT (relative to CC) was 2.25 (95% confidence interval, CI, 1.40–3.62, *p* = 0.0034) under the co-dominant model and 2.27 (95% CI, 1.41–3.66, *p* = 0.0013) under the over-dominant model. Under the dominant model, the odds ratio revealed that individuals possessing the T allele (CT+TT) were 2.17 times more likely to be sprint/power athletes (95% CI 1.35–3.49, *p* = 0.0021) compared with CC homozygotes. There were no associations between endurance athletes (Table 3) or those competing in mixed sport disciplines (Table 4). When sport discipline and athletic performance were considered (Table 5), significant associations in the same directions were found only in the sprint/power group for highly elite athletes (co-dominant, dominant, and over-dominant model) and for sub-elite athletes (over-dominant model). In the highly elite athletes, the strength of association measured by OR was approximately two times greater than in all sprint/power athletes (Table 6). A detailed summary of associations stratified by athletic performance is presented in Supplementary Tables S1–S8.

Four haplotypes were reconstructed (Table 7). In all individuals except one, haplotypes could be assigned unambiguously. Two haplotypes, CAA and TAA (rs10497520, rs55837610, rs72648256, respectively), had frequencies greater than 1%, and these haplotypes were considered in the association analysis. These two haplotypes existed as three diplotypes (matched pairs of haplotypes), namely: CAA/CAA, CAA/TAA, and TAA/TAA. The distribution of diplotypes in control individuals and athletes stratified by sport discipline is shown in Supplementary Table S9. The association of diplotypes with athletic status using a generalised linear model is presented in Table 8. As in single SNP analysis, we found an association in the sprint/power athletes (all athletes, highly elite athletes, and sub-elite athletes). Possessing at least one TAA haplotype (dominant model) resulted in an increase in the log-odds ratio by 0.80 (*p* = 0.00146), 1.42 (*p* = 0.003), and 0.77 (*p* = 0.044) for all sprint/power athletes, high elite sprint/power athletes, and sub-elite sprint/power athletes, respectively. This translates into a 123%, 314%, and 116% increase in odds of being a sprint/power athlete, high elite sprint/power athlete, and sub-elite sprint/power athlete, respectively.

## 4. Discussion

Currently, rapid advances in technologies in the field of genomics, such as high-throughput DNA sequencing combined with improved and new methods of analysis, are helping sports and medicine sciences achieve goals towards precision sports medicine and exercise prescription. These novel data are predicted to advance precision sport sciences considerably by facilitating optimally tailored training programs based on an individual’s molecular profile, which includes their genomic information [28,29]. Knowledge regarding the biochemical, physiological, and genetic factors that affect muscle performance is key to achieving success in sports [30].

Titin plays a pivotal role in muscle passive stiffness, flexibility, tension, and strength [6,8]. Therefore, the *TTN* gene may be considered as a promising genetic marker for athletic status, which may underlie differences in the potential to achieve outstanding results in sports competition. However, analysis of the whole gene is demanding due to its complex structure and large size, and only a few studies have been undertaken to clarify its role in the formation of physical performance [20,25,31] and injury risk [26]. Recently, with DNA sequencing becoming widely available, it has been possible to determine the individual polymorphism located in the *TTN* gene that may influence elite athletic status, which was the main aim of our study. Therefore, we selected three polymorphisms: one previously described missense rs10497520 (Lys1201Glu) polymorphism, and due to our WGS results, two novel missense rs55837610 (Ile23902Thr) and rs72648256 (Asp31617Glu) polymorphisms. The first one is localised in the part of the *TTN* gene encoding the titin Z-disc region and the other two in the protein A-band (https://fraternalilab.kcl.ac.uk/TITINdb/search_page/ (accessed on 12 April 2022)).

The experiment revealed that harbouring the specific *TTN* genotypes is significantly associated with athletic performance and athletes’ competition level in the Caucasian population. Specifically, we found that the rs10497520 CT and TT genotypes were significantly more frequent among the sprint/power athlete group. The T allele carriers were over 2.17 times more likely to be sprint/power athletes compared with the CC homozygotes, indicating that the T allele is favourable for sprint/power performance. When competition level was considered, we found that the likelihood of having the TT genotype was higher for the highly elite power athletes (co-dominant, dominant, and over-dominant model) and for sub-elite athletes (over-dominant model). Furthermore, these results were confirmed in the complex haplotype analysis and demonstrated that harbouring at least one TAA haplotype (rs10497520, rs55837610, or rs72648256, respectively) may be favourable for achieving success in sprint/power sports. In particular, the chance of being a sprint/power athlete, high elite sprint/power athlete, or sub-elite sprint/power athlete was 123%, 314%, and 116% higher, respectively. These findings provide support for the potential influential role of the *TTN* rs10497520 polymorphism in determining elite athletic status in sprint/power athletes, and point to the T allele as a beneficial factor for sprint/power performance, which constitutes the most important finding of this study. This knowledge may potentially be used during screening to identify young athletes’ predisposition for a certain type of sport, which may be a vital component of many sports programs and would also be useful to guide children towards the most suited athletic discipline. For trainers, it may serve as an additional source of detailed information, helpful in the personalisation of training methods and more efficient management of the sports careers of trained athletes. However, it needs to be highlighted that physical performance is a very complex trait, which is pre-determined by inherited traits against the degree of influence by environmental factors (training, diet, motivation, development opportunities, and health conditions). Stebbings et al. suggested that *TTN* variants, specifically rs10497520, may contribute to the variability within titin isoform expression in muscle tissue [20]. The length of skeletal muscle titin is directly related to sarcomere elongation, elasticity, and stiffness. This is associated with the I-band region variations, especially in different lengths of the PEVEK region (rich in proline, glutamic acid, valine, and lysine residues) and different numbers of immunoglobulin (Ig) domains. The longer isoforms are more elastic, while the shorter isoforms are more rigid, thus, increasing passive stiffness and improving motor control [6,16,17,18]. Therefore, *TTN* gene variants may result in the production of shorter or longer isoforms, which alter contractile characteristics [8,20]. Additionally, due to the selected variants’ localisation, the missense rs10497520 polymorphism may lead to the production of normal length isoforms but is poorly attached to the Z-disc, which may impair the mechanical stability of sarcomeres. However, the missense rs55837610 and rs72648256 polymorphisms may modify the stabilisation of myosin filaments in the A-band region and, thus, influence sarcomere structure and contraction. These molecular changes may impact (positively or negatively) muscle performance and overall sports skills.

To the best of our knowledge, this is the first study to analyse the association of the *TTN* rs10497520, rs55837610, rs72648256 polymorphisms, either individually or in haplotype combination, with athletic performance and competition level in elite Caucasian athletes. Only rs10497520 was previously analysed in the context of sports predispositions. Therefore, our results cannot be discussed with direct comparisons to other studies. However, the literature includes a genome-wide linkage scan for training-induced changes in submaximal exercise stroke volume (∆SV50) in the HERITAGE Family Study including 483 individuals, demonstrating that *TTN* seems to be a promising candidate gene for human variation in ∆SV50 in the sedentary state and adapting cardiac function to endurance training [31]. Further evidence was provided by combining RNA profiling with single-gene DNA marker association analysis, which indicated that the rs10497520 polymorphism may be linked to the post-training changes in VO_2_max in the selected 473 participants [25]. In a study including 278 Caucasian males, Stebbings et al. examined the relationship between this SNP and muscle fascicle length in recreationally active men, and marathon personal best times in marathon runners. These results showed that the T allele was associated with a shorter length of the skeletal muscle fascicle, which requires less energy to produce a given force and conveys an advantage for marathon-running performance in trained individuals. They suggested that CC homozygotes possess longer vastus lateralis fascicles, which may be associated with successful sprint running performance [20]. Surprisingly, our results revealed that the T allele was associated with sprint/power—not endurance—performance. These inconsistent results confirm that we are only in the early stages of understanding the relationship between *TTN* polymorphisms and physical performance, and further research is needed. During physical activity or sport, the complex structural organisation of skeletal muscle is easily stressed and damaged, leading to frequent injuries. In sports, muscle injuries comprise 10–55% of all injuries [32]. These injuries reduce or even prevent participation in physical or occupational activities for at least several weeks, affecting athletes’ performance and career, team results and financial aspects, as well as having a negative impact on their quality of life. These consequences support the need for improved pre- and post-injury health care. Although they have been relatively well described at the clinical level, the molecular mechanisms causing these injuries still remain mostly unknown, and we are only in the early stages of understanding the associations between genetic variants and the risk of muscle injuries. In the future, genetic studies may be used by clinicians, coaches, and athletes to develop personalised programs for the pre-habilitation of susceptible individuals, or to aid recovery after overloading/injury (personalised medicine). Additionally, the potential biological mechanisms connecting titin with injuries have been described by Perrin et al. [27], thus, it has been assumed that the variants within the *TTN* gene may be also associated with the risk of muscle injuries. It was suggested that rs10497520 CC genotype carriers with longer skeletal muscle fascicles may have a rightward shift in the length–tension relationship and greater optimal joint angles for maximal torque production. Such a shift has been linked to a decreased risk of injury, as a longer optimal skeletal muscle length would mean that less of the muscle’s functional range would be along the more unstable descending limb of the length–tension curve. Conversely, T allele carriers may be susceptible to injuries [20,33]. The importance of the *TTN* gene for muscle properties and injury risk was confirmed by Vera et al., who showed the association between connective tissue disorders (CTDs) and genetic variants within this gene [26]. In the future, knowledge of the *TTN* genotype in populations at higher injury risk, such as athletes and physically active people, may help to predict the consequences of physical activity and tailored training interventions specific to the *TTN* genotype [20]. However, further research is required before the information from genetic studies will be able to be used in practice.

The strengths of our study include the exact experimental organisation, particularly in combining DNA sequencing methodology and subsequent advanced bioinformatic assessment with the association of selected *TTN* variants with sport-related phenotypes. It needs to be highlighted that these data are the first effort in applying WGS to determine the significance of novel and previously described allelic variants, genotypes, and haplotypes located in the *TTN* gene related to physical performance and to the achievement of outstanding results in sports competition. The chosen methodology allows the identification of sport-related variants in both coding and non-coding regions of the gene. A potential limitation of the present study is population-specific characteristics, such as the highly homogenous European background of the cohort. Thus, we were unable to perform an analysis by ethnicity. Additionally, none, or a low number, of some homozygotes in the Caucasian population required us to analyse the homozygotes in combination with the heterozygotes and, thus, may be potential confounders in the analyses.

## 5. Conclusions

This study provides evidence for the association between *TTN* variants and sports skills, which may underline differences in the potential to be an elite athlete. We demonstrated that harbouring at least one of the rs10497520 T alleles, either individually or in haplotype combination, increases the chance of being an elite sprint/power athlete, indicating that the T allele is favourable for sprint/power performance. In conclusion, we believe that the *TTN* gene may be a promising genetic marker for athletic performance and competition level. In the future, this knowledge may potentially be used during screening to find new sports talents, as well as an additional source of detailed information helpful in the personalisation of training methods and more efficient management of the sports careers of trained athletes. However, far more studies are required to establish the role of these variants and their implications for human health, physical activity, adaptive changes in muscles in response to training, and injury risk.

## Figures and Tables

**Table 1:** Single SNP association analysis for cases (all sports) and controls.

SNP	Model	Control (n = 403)	Sport (n = 348)	OR	95% CI	*p* *
rs10497520	Co-dominant					
	C/C	320 (79.4)	258 (74.1)	1		0.200/ 0.169
	C/T	80 (19.9)	88 (25.3)	1.36	0.97–1.93	
	T/T	3 (0.7)	2 (0.6)	0.83	0.14–4.99	
	Dominant					
	C/C	320 (79.4)	258 (74.1)	1		0.088/ 0.075
	C/T-T/T	83 (20.6)	90 (25.9)	1.34	0.96–1.89	
	Recessive					
	C/C-C/T	400 (99.3)	346 (99.4)	1		0.775/ 0.732
	T/T	3 (0.7)	2 (0.6)	0.77	0.13–4.64	
	Over-dominant					
	C/C-T/T	323 (80.1)	260 (74.7)	1		0.075/ 0.062
	C/T	80 (19.9)	88 (25.3)	1.37	0.97–1.93	
rs55837610	Co-dominant					
	A/A	399 (99.0)	344 (98.9)	1		0.835/ 0.905
	A/G	4 (1.0)	4 (1.1)	1.16	0.29–4.67	
rs72648256	Co-dominant					
	A/A	401 (99.5)	346 (99.4)	1		0.883/ 0.791
	A/T	2 (0.5)	2 (0.6)	1.16	0.16–8.27	

* raw *p* values and sex adjusted (after slash).

**Table 2:** Single SNP association analysis for cases (sprint/power sports) and controls.

SNP	Model	Control (n = 403)	Sport (n = 100)	OR	95% CI	*p* *
rs10497520	Co-dominant					
	C/C	320 (79.4)	64 (64.0)	1		0.0025/ 0.0034
	C/T	80 (19.9)	36 (36.0)	2.25	1.40–3.62	
	T/T	3 (0.7)	0 (0)	0		
	Dominant					
	C/C	320 (79.4)	64 (64.0)	1		0.0017/ 0.0021
	C/T-T/T	83 (20.6)	36 (36.0)	2.17	1.35–3.49	
	Recessive					
	C/C-C/T	400 (99.3)	100 (100.0)	1		1.0/ 0.279
	T/T	3 (0.7)	0 (0)	0		
	Over-dominant					
	C/C-T/T	323 (80.1)	64 (64.0)	1		0.00095/ 0.0013
	C/T	80 (19.9)	36 (36.0)	2.27	1.41–3.66	
rs55837610	Co-dominant					
	A/A	399 (99.0)	98 (98.0)	1		0.437/ 0.438
	A/G	4 (1.0)	2 (2.0)	2.04	0.37–11.27	
rs72648256	Co-dominant					
	A/A	401 (99.5)	100 (100.0)	1		1.0/ 0.236
	A/T	2 (0.5)	0 (0)	0		

* raw *p* values and sex adjusted (after slash).

**Table 3:** Single SNP association analysis for cases (endurance sports) and controls.

SNP	Model	Control (n = 403)	Sport (n = 108)	OR	95% CI	*p* *
rs10497520	Co-dominant					
	C/C	320 (79.4)	92 (85.2)	1		0.345/ 0.380
	C/T	80 (19.9)	15 (13.9)	0.65	0.36–1.19	
	T/T	3 (0.7)	1 (0.9)	1.16	0.12–11.28	
	Dominant					
	C/C	320 (79.4)	92 (85.2)	1		0.167/ 0.190
	C/T-T/T	83 (20.6)	16 (14.8)	0.67	0.37–1.20	
	Recessive					
	C/C-C/T	400 (99.3)	107 (99.1)	1		0.852/ 0.838
	T/T	3 (0.7)	1 (0.9)	1.25	0.13–12.10	
	Over-dominant					
	C/C-T/T	323 (80.1)	93 (86.1)	1		0.146/ 0.167
	C/T	80 (19.9)	15 (13.9)	0.65	0.36–1.18	
rs55837610	Co-dominant					
	A/A	399 (99.0)	107 (99.1)	1		0.950/ 0.838
	A/G	4 (1.0)	1 (0.9)	0.93	0.10–8.43	
rs72648256	Co-dominant					
	A/A	401 (99.5)	108 (100.0)	1		1.0/ 0.195
	A/T	2 (0.5)	0 (0)	0		

* raw *p* values and sex adjusted (after slash).

**Table 4:** Single SNP association analysis for cases (mixed-sports) and controls.

SNP	Model	Control (n = 403)	Sport (n = 140)	OR	95% CI	*p* *
rs10497520	Co-dominant					
	C/C	320 (79.4)	102 (72.9)	1	1	0.276/ 0.397
	C/T	80 (19.9)	37 (26.4)	1.45	0.93–2.27	
	T/T	3 (0.7)	1 (0.7)	1.05	0.11–10.16	
	Dominant					
	C/C	320 (79.4)	102 (72.9)	1	1	0.114/ 0.181
	C/T-T/T	83 (20.6)	38 (27.1)	1.44	0.92–2.24	
	Recessive					
	C/C-C/T	400 (99.3)	139 (99.3)	1	1	0.971/ 0.973
	T/T	3 (0.7)	1 (0.7)	0.96	0.10–9.30	
	Over-dominant					
	C/C-T/T	323 (80.1)	103 (73.6)	1	1	0.108/ 0.174
	C/T	80 (19.9)	37 (26.4)	1.45	0.93–2.27	
rs55837610	Co-dominant					
	A/A	399 (99.0)	139 (99.3)	1	1	0.760/ 0.665
	A/G	4 (1.0)	1 (0.7)	0.72	0.08–6.48	
rs72648256	Co-dominant					
	A/A	401 (99.5)	138 (98.6)	1	1	0.299/ 0.275
	A/T	2 (0.5)	2 (1.4)	2.91	0.41–20.83	

* raw *p* values and sex adjusted (after slash).

**Table 5:** Odds ratios with 95% confidence interval for comparisons of athletes (stratified by sport discipline and athletic performance) and controls.

Group	SNP	Model		High Elite		Elite		Sub-Elite	
				OR	95% CI	OR	95% CI	OR	95% CI
Sprint/power	rs10497520	Co-dominant	C/T vs. C/C	4.44	1.75–11.30 *	1.62	0.82–3.24	2.26	1.10–4.66
			T/T vs. C/C	0		0		0	
		Dominant		4.28	1.69–10.88 *	1.57	0.79–3.12	2.18	1.06–4.48
		Recessive		0		0		0	
		Over-dominant		4.49	1.76–11.41 *	1.64	0.82–3.27	2.28	1.11–4.70 *
	rs55837610	Co-dominant	A/G vs. A/A	0		2.27	0.25–20.73	2.85	0.31–26.2
	rs72648256	Co-dominant	A/T vs. A/A	0		0		0	
Endurance	rs10497520	Co-dominant	C/T vs. C/C	1.07	0.34–3.30	0.82	0.37–1.82	0.32	0.10–1.05
			T/T vs. C/C	0		0		2.81	0.28–27.66
		Dominant		1.03	0.33–3.18	0.79	0.36–1.76	0.41	0.14–1.17
		Recessive		0		0		3.25	0.33–31.98
		Over-dominant		1.08	0.35–3.33	0.83	0.37–1.84	0.31	0.09–1.03
	rs55837610	Co-dominant	A/G vs. A/A	0		2.17	0.24–19.82	0	
	rs72648256	Co-dominant	A/T vs. A/A	0		0		0	
Mixed-sports	rs10497520	Co-dominant	C/T vs. C/C	2.18	0.78–6.08	1.28	0.68–2.40	1.45	0.78–2.71
			T/T vs. C/C	0		0		2.42	0.25–23.82
		Dominant		2.10	0.76–5.85	1.23	0.66–2.31	1.49	0.81–2.74
		Recessive		0		0		2.22	0.23–21.71
		Over-dominant		2.20	0.79–6.13	1.29	0.69–2.42	1.44	0.77–2.67
	rs55837610	Co-dominant	A/G vs. A/A	0		0		1.66	0.18–15.13
	rs72648256	Co-dominant	A/T vs. A/A	0		6.68	0.92–48.34	0	

* Significant (*p* < 0.05) after controlling for sex.

**Table 6:** Single SNP association analysis for cases (sprint/power sports, high elite) and controls.

SNP	Model	Control	Sport (n = 19)	OR	95% CI	*p* *
rs10497520	Co-dominant					
	C/C	320 (79.4)	9 (47.4)	1	1	0.009694/ 0.009898
	C/T	80 (19.9)	10 (52.6)	4.44	1.75–11.30	
	T/T	3 (0.7)	0 (0)	0	0	
	Dominant					
	C/C	320 (79.4)	9 (47.4)	1	1	0.002793/ 0.003333
	C/T-T/T	83 (20.6)	10 (52.6)	4.28	1.69–10.88	
	Recessive					
	C/C-C/T	400 (99.3)	19 (100.0)	1	1	1.0/ 0.617126
	T/T	3 (0.7)	0 (0)	0	0	
	Over-dominant					
	C/C-T/T	323 (80.1)	9 (47.4)	1	1	0.002092/ 0.002584
	C/T	80 (19.9)	10 (52.6)	4.49	1.76–11.41	
rs55837610	Co-dominant					
	A/A	399 (99.0)	19 (100.0)	1	1	1.0/ 0.5278
	A/G	4 (1.0)	0 (0)	0	0	
rs72648256	Co-dominant					
	A/A	401 (99.5)	19 (100.0)	1	1	1.0/ 0.5861
	A/T	2 (0.5)	0 (0)	0	0	

* raw *p* values and sex adjusted (after slash).

**Table 7:** Frequency of haplotypes.

rs10497520	rs55837610	rs72648256	Frequency
C	A	A	0.87350
C	A	T	0.00266
C	G	A	0.00533
T	A	A	0.11851

**Table 8:** Association of diplotypes with athletic status (case vs. control) using a generalised linear model, stratified by sport discipline and athletic performance, and adjusted for sex.

Group	All	High Elite	Elite	Sub-Elite
Sprint/power	0.80 (0.00146)	1.42 (0.003)	0.46 (0.197)	0.77 (0.044)
Endurance	−0.41 (0.191)	−0.03 (0.961)	−0.24 (0.564)	−0.97 (0.079)
Mixed	0.34 (0.158)	0.64 (0.231)	0.21 (0.530)	0.37 (0.237)

Model coefficients and *p* values in brackets.

## Data Availability

The data presented in this study are available on request from the corresponding author. The data are not publicly available due to privacy/ethical restrictions.

## Supplementary Materials

The following supporting information can be downloaded at: https://www.mdpi.com/article/10.3390/ijerph191610173/s1.

## References

[1] Huxley H., Hanson J. (1954). Changes in the Cross-striations of muscle during contraction and stretch and their structural interpretation. Nature.

[2] Huxley A.F., Niedergerke R. (1954). Structural changes in muscle during contraction: Interference microscopy of living muscle fibres. Nature.

[3] Maruyama K. (1976). Connectin, an elastic protein from myofibrils. J. Biochem..

[4] Maruyama K. (1997). Connectin/titin, giant elastic protein of muscle. FASEB J..

[5] Wang K., McClure J., Tu A. (1979). Titin: Major myofibrillar components of striated muscle. Proc. Natl. Acad. Sci. USA.

[6] Chauveau C., Rowell J., Ferreiro A. (2014). A rising titan: *TTN* review and mutation update. Hum. Mutat..

[7] Adewale A.O., Ahn Y.H. (2021). Titin N2A domain and its interactions at the sarcomere. Int. J. Mol. Sci..

[8] Leońska-Duniec A., Maciejewska-Skrendo A. (2021). *TTN* Gene’s Variants as Potential Markers Associated with Muscle Tissue’s Disfunctions and Physical Performance. Acta Kinesiol..

[9] Monroy J.A., Powers K.L., Gilmore L.A., Uyeno T.A., Lindstedt S.L., Nishikawa K.C. (2012). What is the role of titin in active muscle?. Exerc. Sport Sci. Rev..

[10] Lieber R.L., Roberts T.J., Blemker S.S., Lee S.S.M., Herzog W. (2017). Skeletal muscle mechanics, energetics and plasticity. J. Neuroeng. Rehabil..

[11] Linke W.A. (2018). Titin Gene and Protein Functions in Passive and Active Muscle. Annu. Rev. Physiol..

[12] Granzier H., Labeit S. (2007). Structure-function relations of the giant elastic protein titin in striated and smooth muscle cells. Muscle Nerve..

[13] Linke W.A., Krüger M. (2010). The giant protein titin as an integrator of myocyte signaling pathways. Physiology.

[14] Linke W.A., Popov V.I., Pollack G.H. (1994). Passive and active tension in single cardiac myofibrils. Biophys. J..

[15] Eckels E.C., Tapia-Rojo R., Rivas-Pardo J.A., Fernández J.M. (2018). The Work of Titin Protein Folding as a Major Driver in Muscle Contraction. Annu. Rev. Physiol..

[16] Freiburg A., Trombitas K., Hell W., Cazorla O., Fougerousse F., Centner T., Kolmerer B., Witt C., Beckmann J.S., Gregorio C.C. (2000). Series of exon-skipping events in the elastic spring region of titin as the structural basis for myofibrillar elastic diversity. Circ. Res..

[17] Labeit S., Kolmerer B. (1995). Titins: Giant proteins in charge of muscle ultrastructure and elasticity. Science.

[18] Wang K., Mccarter R., Wright J., Beverly J., Ramirez-Mitchell R. (1991). Regulation of skeletal muscle stiffness and elasticity by titin isoforms: A test of the segmental extension model of resting tension. Proc. Natl. Acad. Sci. USA.

[19] Fry A.C., Staron R.S., James C.B.L., Hikida R.S., Hagerman F.C. (1997). Differential titin isoform expression in human skeletal muscle. Acta Physiol. Scand..

[20] Stebbings G.K., Williams A.G., Herbert A.J., Lockey S.J., Heffernan S.M., Erskine R.M., Morse C.I., Day S.H. (2018). *TTN* genotype is associated with fascicle length and marathon running performance. Scand. J. Med. Sci. Sport.

[21] Lewinter M.M., Granzier H.L. (2013). Titin is a major human disease gene. Circulation.

[22] Tharp C.A., Haywood M.E., Sbaizero O., Taylor M.R.G., Mestroni L. (2019). The Giant Protein Titin’s Role in Cardiomyopathy: Genetic, Transcriptional, and Post-translational Modifications of *TTN* and Their Contribution to Cardiac Disease. Front. Physiol..

[23] Savarese M., Valipakka S., Johari M., Hackman P., Udd B. (2020). Is Gene-Size an Issue for the Diagnosis of Skeletal Muscle Disorders?. J. Neuromuscul. Dis..

[24] Kellermayer D., Smith J.E., Granzier H. (2019). Titin mutations and muscle disease. Pflugers. Arch..

[25] Timmons J.A., Knudsen S., Rankinen T., Koch L.G., Sarzynski M., Jensen T., Keller P., Scheele C., Vollaard N., Nielsen S. (2010). Using molecular classification to predict gains in maximal aerobic capacity following endurance exercise training in humans. J. Appl. Physiol..

[26] Vera A.M., Peterson L., Dong D., Haghshenas V., Yetter T., Delgado D.A., McCulloch P., Varner K.E., Harris J. (2020). High Prevalence of Connective Tissue Gene Variants in Professional Ballet. Am. J. Sports Med..

[27] Perrin C., Nosaka K., Steele J. (2017). Could titin have a role in strain-induced injuries?. J. Sport Health Sci..

[28] Tanisawa K., Wang G., Seto J., Verdouka I., Twycross-Lewis R., Karanikolou A., Tanaka M., Borjesson M., Di Luigi L., Dohi M. (2020). Sport and exercise genomics: The FIMS 2019 consensus statement update. Br. J. Sports Med..

[29] Switala K., Leonska-Duniec A. (2021). Physical activity and gene association with human obesity. Balt. J. Health Phys. Act..

[30] Maciejewska-Skrendo A., Mieszkowski J., Kochanowicz A., Stankiewicz B., Cieszczyk P., Switala K., Gomes de Assis G., Kecler K., Tarnowski M., Sawczuk M. (2019). TNFA expression level changes observed in response to the Wingate Anaerobic Test in non-trained and trained individuals. Balt. J. Health Phys. Act..

[31] Rankinen T., Rice T., Boudreau A., Leon A.S., Skinner J.S., Wilmore J.H., Rao D.C., Bouchard C. (2004). Titin is a candidate gene for stroke volume response to endurance training: The HERITAGE Family Study. Physiol. Genom..

[32] Kaariainen M., Jarvinen T., Jarvinen M., Rantanen J., Kalimo H. (2000). Relation between myofibers and connective tissue during muscle injury repair. Scand. J. Med. Sci. Sports.

[33] Brughelli M., Cronin J. (2007). Altering the length-tension relationship with eccentric exercise: Implications for performance and injury. Sports Med..

