# Supplementary material for: TTN Variants Are Associated with Physical Performance and Provide Potential Markers for Sport-Related Phenotypes
Source: Int J Environ Res Public Health. 2022 Aug 17;19(16):10173. doi: 10.3390/ijerph191610173 (PMC9408402; doi:10.3390/ijerph191610173)
Supplement: Supplementary file 1 [file ijerph-19-10173-s001.zip › ijerph-1793999-supplementary.pdf]

Supplementary Table S1. Single SNP association analysis for cases (sprint/power sports, elite) and controls

SNP	Model	Control (n=403)	Sport (n=45)	OR (95% CI)	P*
rs10497520	Codominant				
	C/C	320 (79.4)	32 (71.1)	1	0.401/ 0.319
	C/T	80 (19.9)	13 (28.9)	1.62 (0.82-3.24)	
	T/T	3 (0.7)	0 (0)	0	
	Dominant				
	C/C	320 (79.4)	32 (71.1)	1	0.213/ 0.223
	C/T-T/T	83 (20.6)	13 (28.9)	1.57 (0.79-3.12)	
	Recessive				
	C/C-C/T	400 (99.3)	45 (100.0)	1	1.0/ 0.448
	T/T	3 (0.7)	0 (0)	0	
	Overdominant				
	C/C-T/T	323 (80.1)	32 (71.1)	1	0.172/ 0.184
	C/T	80 (19.9)	13 (23.9)	1.64 (0.82-3.27)	
rs55837610	Codominant				
	A/A	399 (99.0)	44 (97.8)	1	0.505/
	A/G	4 (1.0)	1 (2.2)	2.27 (0.25-20.73)	0.573
rs72648256	Codominant				
	A/A	401 (99.5)	45 (100.0)	1	1.0/
	A/T	2 (0.5)	0 (0)	0 (0)	0.419

* raw P values and sex adjusted (after slash)

Supplementary Table S2. Single SNP association analysis for cases (sprint/power sports, subelite) and controls

SNP	Model	Control (n=403)	Sport (n=36)	OR (95% CI)	P*
rs10497520	Codominant				
	C/C	320 (79.4)	23 (63.9)	1	0.072/ 0.113
	C/T	80 (19.9)	13 (36.1)	2.26 (1.10-4.66)	
	T/T	3 (0.7)	0 (0)	0	
	Dominant				
	C/C	320 (79.4)	23 (63.9)	1	0.041/ 0.057
	C/T-T/T	83 (20.6)	13 (36.1)	2.18 (1.06-4.48)	
	Recessive				
	C/C-C/T	400 (99.3)	36 (100.0)	1	1.0/ 0.506
	T/T	3 (0.7)	0 (0)	0	
	Overdominant				
	C/C-T/T	323 (80.1)	23 (63.9)	1	0.031/ 0.045
	C/T	80 (19.9)	13 (36.1)	2.28 (1.11-4.70)	
rs55837610	Codominant				
	A/A	399 (99.0)	35 (97.2)	1	0.405/
	A/G	4 (1.0)	1 (2.8)	2.85 (0.31-26.2)	0.362
rs72648256	Codominant				
	A/A	401 (99.5)	36 (100.0)	1	1.0/
	A/T	2 (0.5)	0 (0)	0	0.434

* raw P values and sex adjusted (after slash)

Supplementary Table S3. Single SNP association analysis for cases (endurance sports, high elite) and controls

SNP	Model	Control (n=402)	Sport (n=19)	OR (95% CI)	P*
rs10497520	Codominant				
	C/C	320 (79.4)	15 (78.9)	1	1.0/ 0.885
	C/T	80 (19.9)	4 (21.1)	1.07 (0.34-3.30)	
	T/T	3 (0.7)	0 (0)	0	
	Dominant				
	C/C	320 (79.4)	15 (78.9)	1	0.962/
	C/T-T/T	83 (20.6)	4 (21.1)	1.03 (0.33-3.18)	0.986
	Recessive				
	C/C-C/T	400 (99.3)	19 (100.0)	1	1.0/
	T/T	3 (0.7)	0 (0)	0	0.621
	Overdominant				
	C/C-T/T	323 (80.1)	15 (78.9)	1	0.899/
	C/T	80 (19.9)	4 (21.1)	1.08 (0.35-3.33)	0.959
rs55837610	Codominant				
	A/A	399 (99.0)	19 (100.0)	1	1.0/
	A/G	4 (1.0)	0 (0)	0	0.525
rs72648256	Codominant				
	A/A	401 (99.5)	19 (100.0)	1	1.0/
	A/T	2 (0.5)	0 (0)	0	0.573

* raw P values and sex adjusted (after slash)

Supplementary Table S4. Single SNP association analysis for cases (endurance sports, elite) and controls

SNP	Model	Control (n=403)	Sport (n=47)	OR (95% CI)	P*
rs10497520	Codominant				
	C/C	320 (79.4)	39 (83.1)	1	0.787/ 0.661
	C/T	80 (19.9)	8 (17.0)	0.82 (0.37-1.82)	
	T/T	3 (0.7)	0 (0)	0	
	Dominant				
	C/C	320 (79.4)	39 (83.0)	1	0.556/
	C/T-T/T	83 (20.6)	8 (17.0)	0.79 (0.36-1.76)	0.548
	Recessive				
	C/C-C/T	400 (99.3)	47 (100.0)	1	1.0/
	T/T	3 (0.7)	0 (0)	0	0.456
	Overdominant				
	C/C-T/T	323 (80.1)	39 (83.1)	1	0.638/
	C/T	80 (19.9)	8 (17.0)	0.83 (0.37-1.84)	0.616
rs55837610	Codominant				
	A/A	399 (99.0)	46 (97.9)	1	0.526/
	A/G	4 (1.0)	1 (2.1)	2.17 (0.24-19.82)	0.622
rs72648256	Codominant				
	A/A	401 (99.5)	47 (100.0)	1	1.0/
	A/T	2 (0.5)	0 (0)	0	0.368

* raw P values and sex adjusted (after slash)

Supplementary Table S5. Single SNP association analysis for cases (endurance sports, subelite) and controls

SNP	Model	Control (n=403)	Sport (n=42)	OR (95% CI)	P*
rs10497520	Codominant				
	C/C	320 (79.4)	38 (90.5)	1	0.061/ 0.056
	C/T	80 (19.9)	3 (7.1)	0.32 (0.10-1.05)	
	T/T	3 (0.7)	1 (2.4)	2.81 (0.28-27.66)	
	Dominant				
	C/C	320 (79.4)	38 (90.5)	1	0.063/
	C/T-T/T	83 (20.6)	4 (9.5)	0.41 (0.14-1.17)	0.058
	Recessive				
	C/C-C/T	400 (99.3)	41 (97.6)	0	0.363/
	T/T	3 (0.7)	1 (2.4)	3.25 (0.33-31.98)	0.364
	Overdominant				
	C/C-T/T	323 (80.1)	39 (92.9)	1	0.026/
	C/T	80 (19.9)	3 (7.1)	0.31 (0.09-1.03)	0.024
rs55837610	Codominant				
	A/A	399 (99.0)	42 (100.0)	1	1.0/
	A/G	4 (1.0)	0 (0)	0	0.350
rs72648256	Codominant				
	A/A	401 (99.5)	42 (100.0)	1	1.0/
	A/T	2 (0.5)	0 (0)	0	0.380

* raw P values and sex adjusted (after slash)

Supplementary Table S6. Single SNP association analysis for cases (mixed-sports, high elite) and controls

SNP	Model	Control (n=403)	Sport (n=17)	OR (95% CI)	P*
rs10497520	Codominant				
	C/C	320 (79.4)	11 (64.7)	1	0.234/ 0.411
	C/T	80 (19.9)	6 (35.3)	2.18 (0.78-6.08)	
	T/T	3 (0.7)	0 (0)	0	
	Dominant				
	C/C	320 (79.4)	11 (64.7)	1	0.172/
	C/T-T/T	83 (20.6)	6 (35.3)	2.10 (0.76-5.85)	0.226
	Recessive				
	C/C-C/T	400 (99.3)	17 (100.0)	1	1.0/
	T/T	3 (0.7)	0 (0)	0	0.657
	Overdominant				
	C/C-T/T	323 (80.1)	11 (64.7)	1	0.148/
	C/T	80 (19.9)	6 (35.3)	2.20 (0.79-6.13)	0.204
rs55837610	Codominant				
	A/A	399 (99.0)	17 (100.0)	1	1.0/
	A/G	4 (1.0)	0 (0)	0	0.538
rs72648256	Codominant				
	A/A	401 (99.5)	17 (100.0)	1	1.0/
	A/T	2 (0.5)	0 (0)	0	0.548

* raw P values and sex adjusted (after slash)

Supplementary Table S7. Single SNP association analysis for cases (mixed-sports, elite) and controls

SNP	Model	Control (n=403)	Sport (n=63)	OR (95% CI)	P*
rs10497520	Codominant				
	C/C	320 (79.4)	47 (75.8)	1	0.674/ 0.567
	C/T	80 (19.9)	15 (24.2)	1.28 (0.68-2.40)	
	T/T	3 (0.7)	0 (0)	0	
	Dominant				
	C/C	320 (79.4)	47 (75.8)	1	0.524/
	C/T-T/T	83 (20.6)	15 (24.2)	1.23 (0.66-2.31)	0.589
	Recessive				
	C/C-C/T	400 (99.3)	62(100.0)	1	1.0/
	T/T	3 (0.7)	0 (0)	0	0.392
	Overdominant				
	C/C-T/T	323 (80.1)	47 (75.8)	1	0.438/
	C/T	80 (19.9)	15 (24.2)	1.29 (0.69-2.42)	0.511
rs55837610	Codominant				
	A/A	399 (99.0)	62(100.0)	1	1.0/
	A/G	4 (1.0)	0 (0)	0	0.267
rs72648256	Codominant				
	A/A	401 (99.5)	60 (96.8)	1	0.077/
	A/T	2 (0.5)	2 (3.2)	6.68 (0.92-48.34)	0.069

* raw P values and sex adjusted (after slash)

Supplementary Table S8. Single SNP association analysis for cases (mixed-sports, subelite) and controls

SNP	Model	Control (n=403)	Sport (n=61)	OR (95% CI)	P*
rs10497520	Codominant				
	C/C	320 (79.4)	44 (72.1)	1	0.418/ 0.484
	C/T	80 (19.9)	16 (26.2)	1.45 (0.78-2.71)	
	T/T	3 (0.7)	1 (1.6)	2.42 (0.25-23.82)	
	Dominant				
	C/C	320 (79.4)	44 (72.1)	1	0.210/
	C/T-T/T	83 (20.6)	17 (27.9)	1.49 (0.81-2.74)	0.258
	Recessive				
	C/C-C/T	400 (99.3)	60 (98.4)	1	0.522/
	T/T	3 (0.7)	1 (1.6)	2.22 (0.23-21.71)	0.538
	Overdominant				
	C/C-T/T	323 (80.1)	45 (73. 8)	1	0.264/
	C/T	80 (19.9)	16 (26.2)	1.44 (0.77-2.67)	0.318
rs55837610	Codominant				
	A/A	399 (99.0)	60 (98.4)	1	0.668/
	A/G	4 (1.0)	1 (1.6)	1.66 (0.18-15.13)	0.742
rs72648256	Codominant				
	A/A	401 (99.5)	61 (100.0)	1	1.0/
	A/T	2 (0.5)	0 (0)	0	0.362

* raw P values and sex adjusted (after slash)

Supplementary Table S9. Diplotype count and frequency per group

Group	Diplotype	N	Total	Frequency
Control	CAA/CAA	315	397	79.3
Control	CAA/TAA	79	397	19.9
Control	TAA/TAA	3	397	0.8
Endurance	CAA/CAA	91	107	85.0
Endurance	CAA/TAA	15	107	14.0
Endurance	TAA/TAA	1	107	0.9
Mix	CAA/CAA	99	137	72.3
Mix	CAA/TAA	37	137	27.0
Mix	TAA/TAA	1	137	0.7
Power	CAA/CAA	62	98	63.3
Power	CAA/TAA	36	98	36.7
